# Acute effects of thermally processed pili (*Canarium ovatum*, Engl.) pomace drink on plasma antioxidant and polyphenol status in humans

**Published:** 2017

**Authors:** Elizabeth Hashim Arenas, Trinidad Palad Trinidad

**Affiliations:** 1 *Department of Food Technology, College of Education, University of Santo Tomas, Espana, Manila, 1015, Philippines*; 2 *The Graduate School, University of Santo Tomas, Espana, Manila, 1015, Philippines*

**Keywords:** Polyphenols, Plasma, Antioxidant capacity, Canarium ovatum

## Abstract

**Objective:**

Pili (*Canarium ovatum, *Engl*.*) pomace is an underutilized agricultural waste that possesses great potential to be regarded as a functional food ingredient. The aim of this study was to measure the polyphenol content and antioxidant activity of pili pomace drink and determine the influence of heating on these parameters. Moreover, it sought to assess the acute effects of thermally processed pili pomace drink on plasma antioxidant and polyphenol status in humans.

**Material and Methods:**

Ten healthy adults received a single dose (130 ml) of pili pomace drink following an overnight fasting, and blood was collected at 0, 30, 60, 120 and 240 min after ingestion of pili pomace. Plasma total polyphenol content was measured using Folin-Ciocalteu method, while total antioxidant capacity (TAC) was determined using ferric reducing antioxidant power (FRAP) assay in uricase-treated and untreated plasma samples.

**Results:**

Significant changes in plasma antioxidant and polyphenol levels were observed, reaching maximum levels at 120 and between 30 – 60 min, respectively (p<0.05). Both plasma polyphenols and TAC remained significantly above baseline values throughout the entire test period (p<0.05).

**Conclusion:**

Results raised the possibility that an acute consumption of this phenolic-rich pili pomace drink may enhance plasma antioxidant and polyphenol status in humans. Future studies on other unidentified metabolites from pili pomace that may have enhanced the antioxidant activity of plasma should be done.

## Introduction

Polyphenols derived from fruits and beverages are the most abundant sources of dietary antioxidants. These antioxidant compounds protect the body against oxidative damage that are known to cause degenerative diseases. In fact, the preventive role of polyphenol-rich diet on diseases has been suggested in epidemiological studies (Scalbert and Williamson, 2000 ▶). Recently, it was reported that high polyphenol intake reduced the risk of mortality (Zamora-Ros et al., 2013 ▶). Previous studies have demonstrated that polyphenol-rich beverages enhanced human plasma antioxidant capacity immediately after consumption. Moreover, this effect has been ascribed to the increase in polyphenol plasma concentration (Duthie et al., 1998 ▶; Serafini et al., 1998 ▶; Ghiselli et al., 2000a ▶; Leenen et al., 2000 ▶). Thus, an “acute” study could be a suitable approach for the preliminary screening of *in vivo* effects of food on total antioxidant capacity (Serafini and Del Rio, 2004 ▶). 

*Canarium ovatum* Engl., locally called “pili”, is a tropical nut-bearing tree. It is only in the Philippines where it is commercially grown and processed. This tree is mainly cultivated in the Bicol region of Southern Luzon, and the provinces of Sorsogon, Albay and Camarines Sur are the top producers. Oil extracted from the fruit pulp can be used for cooking and production of soaps and cosmetics (Coronel, 1983 ▶). Production of pili pulp oil is accomplished by mechanical press. This process generates a solid waste residue or pomace, which is composed of peel and fibrous pulp. Currently, the local pili industry faces limited options for the recycling of this waste product. 

Pili pomace is an unexploited source of phytonutrients. Based on earlier *in vitro *digestion studies in our laboratory, this fruit residue was found to be a potentially bioavailable source of polyphenol antioxidants. However, *in vitro* experiments are not adequate to reveal the biological effects of pili pomace. Evidence from an *in vivo* human study can provide a better insight on its possible health benefits. The purpose of this study was to determine the short-term effects of pili pomace intake on the plasma antioxidant and polyphenol status in humans.

## Materials and Methods

**Preparation of experimental beverage**

Pili pomace from a local pili pulp oil producer in Sorsogon City, Philippines was used in this study. Samples were freeze-dried and ground into powder. Freeze-dried sample (10 g) was weighed in clean glass jars. Freshly boiled distilled water (200 ml) was poured into bottles and the mixture was stirred to disperse the fruit pomace. Bottles were then sealed and processed under 10 lbs pressure for 20 min. The product was cooled at ambient temperature and stored at refrigerated temperature for future analysis. This cold storage likewise facilitated sedimentation, wherein dispersed solid particulates settled at the bottom. This process separated the suspension into two distinct phases – solid (residue) and liquid (supernatant). Both phases were examined for polyphenol content and antioxidant capacity. To investigate the effect of heat on the polyphenol content and antioxidant capacity of pili pomace suspension, an unheated sample of the same composition was analyzed along with the processed suspension. 

**Analytical procedures**

The liquid fraction (supernatant) of the suspension was directly used in all assays. Meanwhile, the solid residue was extracted according to a scheme developed by Saura-Calixto et al. (2007). Here, 0.5 g sample was combined with 20 ml acidic ethanol/water (50:50, v/v, pH 2). The mixture was stirred for 1 hr in a shaker and then centrifuged (for 10 min at 2500 *g*). Then, the solid residue was re-extracted with 20 ml of acetone/water (70:30, v/v) under similar conditions. Supernatants obtained after centrifugation were pooled together and used for analysis.

**Polyphenols analysis**

*Total phenolics content*

Sample (0.2 ml) was mixed with 1.5 ml Folin-Ciocalteu reagent (1:10, v/v with distilled water). The mixture was left for 5 min prior to addition of 1.5 ml NaCO_3_ (60g/L). This was followed by a 90-min incubation period. Absorbance was recorded at 725 nm. Results were reported as gallic acid equivalents (GAE) (Velioglu et al., 1998 ▶). 

*Total flavonoids content*

To measure total flavonoids content, 1 ml of the sample was combined with 4 ml of distilled water and 0.3 ml of 5% NaNO_2_ in a 10-ml volumetric flask. After 5 min, 0.3 ml of 10% AlCl_3_ was added. The mixture was incubated for 1 min before addition of 2 ml of 1 M NaOH. Final volume was made up to 10 ml with distilled water. Absorbance was read at 510 nm and results were calculated in terms of catechin equivalents (CE) (Zhishen et al., 1999 ▶). 

*Total monomeric anthocyanin content*

Two sample dilutions were prepared separately using different buffer systems (0.025 M potassium chloride – pH 1.0 and 0.4 M sodium acetate – pH 4.5). These solutions were allowed to equilibrate for 15 min. Absorbance was measured at 510 nm and 700 nm using distilled water as blank. Total monomeric anthocyanin content was expressed as cyanidin 3-glucoside equivalents (C3G) (Giusti and Wrolstad, 2001 ▶).

*Condensed tannins content*

One ml of sample was mixed with 2.5 ml of 1% (w/v) vanillin in ethanol and 2.5 ml of 9 N HCl in ethanol. Absorbance was determined at 500 nm after 20 min incubation at 30^o^C. Tannin levels were expressed as catechin equivalents (CE) (Hajimahmoodi et al., 2008 ▶).

***Determination of antioxidant activity***

*1,1-diphenyl-2-picrylhydrazyl (DPPH) assay*

DPPH radical scavenging activity was assayed by the method of Morales et al. (2008) ▶ with some modifications. To 1 ml aliquot of sample, 2 ml of freshly prepared 0.1 mM DPPH ethanolic solution (2 ml) was added. The resultant mixture was left for 30 min at room temperature. Absorbance was detected at 515 nm.

*2,2’-azinobis-3-ethylbenzothiozoline-6-sulfonic acid (ABTS) assay*

Scavenging activity against ABTS radical was assessed based on the method of Chew et al. (2011) ▶. ABTS radical cation (ABTS^●+^) was generated by mixing equal quantities of 7 mM ABTS stock solution and 2.5 mM K_2_S_2_O_8_. The mixture was left overnight (12-16 hours) in darkness at room temperature before use. This densely coloured ABTS radical solution was then diluted with 70% ethanol to give an absorbance level of 0.70 (+ 0.02) at 734 nm. A 10 ml of diluted ABTS^•+ ^working solution was added to 0.1 ml of sample. Absorbance at 734 nm was read after standing at room temperature for 6 min.

*Ferric reducing antioxidant power (FRAP) assay*

FRAP assay was conducted in accordance with a slightly modified procedure reported by Pulido et al. (2000) ▶. FRAP reagent composed of 5 ml of 10mM TPTZ (2,4,6-tripyridyl-S-triazine) solution, 5 mL of 20mM FeCl_3._6H_2_O_, _and 50 mL of 300mM acetate buffer (pH 3.6) was prepared freshly before analysis. Prior to use, the solution was warmed at 37^o^C. A 0.1 ml aliquot sample was reacted with 3 ml of FRAP reagent. Finally, the mixture was left at 37^o^C in a water bath for 10 min and absorbance was read at 593 nm.

For each antioxidant activity determination, a calibration curve was plotted using known concentrations of Trolox. Results of all antioxidant assays were reported as Trolox equivalents (TE). 

**Study participants**

Ten apparently healthy non-smoking adults (6 females and 4 males), between 25 and 50 years old, were recruited from Food and Nutrition Research Institute – Department of Science and Technology (FNRI-DOST), Taguig City, Philippines. Recruitment was carried out through posters, flyers, and personal communication. None of the female subjects was pregnant or lactating. Subjects were excluded if the following criteria were present: (1) history of any chronic condition or disease; (2) use of medications and dietary supplements; (3) allergy or sensitivity to any food; (4) habitual alcohol consumption; (5) history of drug addiction. Candidates were disqualified if they followed a restricted diet (e.g. vegetarian, vegan or macrobiotic). Volunteers underwent medical examination in order to verify their health status. All participants were found in good health as confirmed by the results of the medical history questionnaire and physical examination. 

Before the start of the study, clearance was secured from the University of Santo Tomas Ethics Review Committee (Protocol No: GS-2014-113-R1) in accordance with the Helsinki Declaration guidelines. Details of the study were carefully explained to the participants and their written informed consent was obtained. Participants were provided with modest compensation and free lunch on the trial day. 

**Study design**

Participants were asked to continue their typical diet, activity level and lifestyle during the weeks before the experiment. Two days before the trial, subjects were instructed to abstain from foods rich in antioxidants and fiber. A list of food products that needed to be excluded from their diet was provided in order to guide the participants. In particular, ingestion of fruits and vegetables or their juices, whole-grain cereal products, chocolate and cocoa derivatives, coffee, tea, wine and nuts was prohibited. Additionally, volunteers were advised to stop taking multivitamin supplements. This step was done in order to partially standardize and limit the intake of antioxidants that would interfere with the evaluation of experimental results. To check adherence to dietary instructions, subjects were asked to fill a food intake record on those two days, indicating the type and amount of food/beverage consumed. Dietary regimen was assessed by a registered dietician. 

The drink used in the study consisted of the supernatant fraction of the processed pili pomace suspension. The supernatant (130 ml) liquid was decanted and collected. This test beverage was served cold to volunteers after a 12-hr fasting period. Subjects were asked to consume the test beverage within 15 min. During the 4-hr experimental period, only water consumption was permitted. Subjects undertook no physical activity but were allowed to read, listen to music or watch television. They were also given free access to bathroom. 

At 0 (baseline), 0.50, 1, 2 and 4 hr after ingestion, blood samples (4 ml) from each subject were collected in heparin vacutainers. Blood collection was performed by a trained and licensed phlebotomist. Clinical trial was supervised by a medical doctor. Immediately after blood extraction, plasma from blood was separated by centrifugation and stored at -80^o^C until analysis.

**Measurement of plasma total polyphenols**

Prior to total phenolics analysis, an acid extraction/hydrolysis and protein precipitation with metaphosphoric acid (MPA) of plasma samples were accomplished, based on the technique reported by Serafini et al. (1998) ▶. This method removes protein and prevents interferences. An aliquot of plasma (500 µl) was mixed with 1 ml 0.75 mol/L MPA. The resultant mixture was vortexed for 3 min and left at 37^o^C for 30 min. Following incubation, the mixture was vortexed for 3 min, then centrifuged at 1500*g* for 10 min. Supernatant was collected, maintained on ice, and kept in the dark. The residue was re-extracted with 1 ml of acetone:water (1:1, v/v) solution and centrifuged at 2700*g* for 10 min. Supernatants were pooled, then filtered using HV 0.45 µm Millipore filter. Determination of total phenolic content was conducted according to the Folin-Ciocalteu assay described above. 

**Measurement of plasma antioxidant capacity**

Plasma antioxidant potential was evaluated using the FRAP method as previously described. For comparison, the ferric-reducing ability of plasma was measured in uricase-treated and untreated samples. Uric acid was removed by addition of uricase according to the method of Modun et al. (2008) ▶. Uricase (Sigma U0880) in phosphate buffer solution (pH 7.5) was added to plasma samples and incubated at 25^o^C for 25 min.

**Statistical analysis**

Experimental data were expressed as mean + standard error of mean (SEM). Paired t-test was used to identify differences between heated and unheated supernatant and residues. Analysis of Variance (ANOVA), followed by Tukey’s HSD test was used to compare plasma antioxidant capacity and total phenol content among time periods. Pearson correlation coefficient was applied to examine association between plasma total phenol concentration and antioxidant capacity. Statistical calculations were performed using SPSS software (version 20.0). Statistical significance was set at p<0.05.

## Results

Characteristics of the subjects are shown in Table 1. Diet information collected from food records were evaluated and summarized in Table 2. All participants followed a low fiber and antioxidant diet. Per—centage of total energy intake from protein and fats were approximately 12 % and 30%, respectively. Carbohydrates contributed to the majority (~60%) of over-all energy intake. 

**Table 1:** Anthropometric and baseline characteristics of volunteers (n = 10).

	**Mean**	**SEM**
**Age (years)**	33.30	2.72
**Height (cm)**	158.31	3.50
**Body mass (kg)**	58.31	2.80
**Body mass index (kg/m** ^2^ **)**	23.22	0.76
**Systolic BP(mmHg)**	109.50	4.37
**Diastolic BP (mmHg)**	74.00	3.40
**Pulse rate (bpm)**	78.70	3.12
**Respiratory rate (cpm)**	19.10	0.28
**Body temperature (** ^o^ **C)**	36.22	0.05

**Table 2:** Estimated nutrient intake of subjects 2 days prior to trial

	**Day 1**	**Day 2**
**Energy (kcal)**	1345.20 +100.63	1541.61 + 163.64
**Carbohydrate (g)**	195.53 + 19.84	215.69 + 32.49
**Protein (g)**	42.18 + 3.91	46.22 + 4.26
**Fat (g)**	43.84 + 6.62	54.90 + 9.13

Mean values + SEM (n=10)

Data revealed that pili pomace suspension contains substantial levels of phenolic compounds including flavonoids, anthocyanins and condensed tannins (Table 3). These compounds are believed to be accountable for the strong antioxidant activity observed in the study (Table 4). Antioxidant activity was evaluated using DPPH, ABTS and FRAP methods. Phenolic antioxidants in pili pomace intake exhibited radical quenching and ferric reducing abilities. Comparison of antioxidant values in the supernatant and residue showed the same order of magnitude – (ABTS > DPPH > FRAP). 

**Figure 1:**
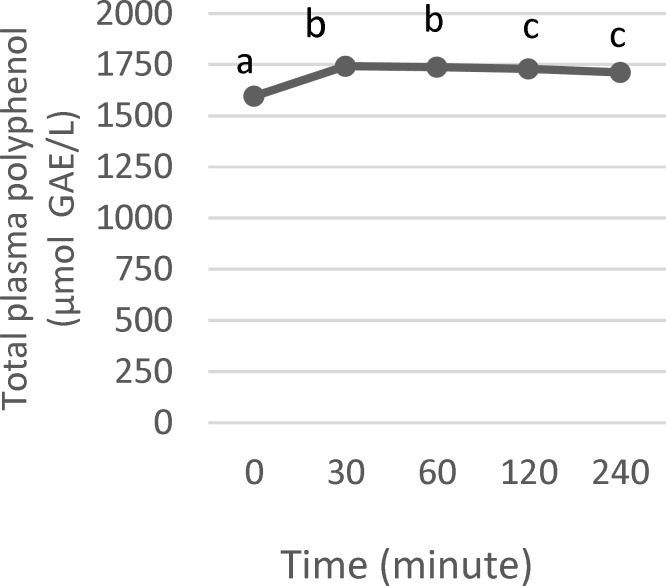
Concentration of total phenolics in human plasma after single intake of pili pomace drink. Das mean+SEM (n=10). Points along the plotted ata line with different subscripts show statistical significance (p< 0.05

As presented in Figure 1, the amount of circulating polyphenols varied over time. Following consumption, polyphenols were detected in the plasma. This evidence implies that pili polyphenols are therefore bioavailable. Compared to baseline, plasma polyphenol concentration significantly increased 30 min after intake and then remained stable within 1 hr (p<0.05). A gradual decrease followed thereafter, but values were still above basal even after 4 hr.

**Figure 2:**
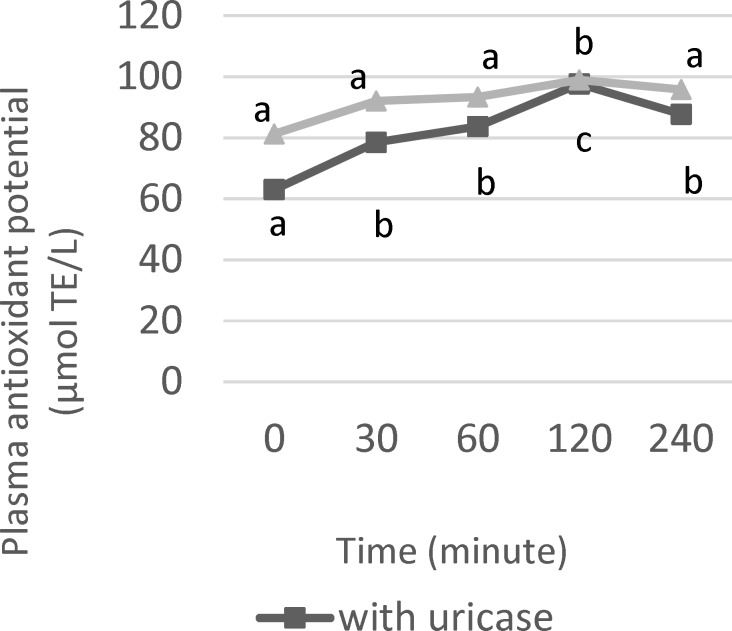
Antioxidant capacity in human plasma after single intake of pili pomace drink. Data as mean+SEM (n=10). Points along the plotted line with different subscripts show statistical significance (p<0.05

The changes in mean plasma TAC are illustrated in Figure 2. The results of the present study support the concept that antioxidant status is modified by dietary treatment particularly by foods high in antioxidant nutrients such as polyphenols. The increase in TAC immediately after ingestion, establishes the direct effect of food antioxidants to *in vivo* antioxidant response. 

TAC values significantly rose as early as 30 min after intake (p<0.05). Likewise, baseline plasma antioxidant capacity levels increased immediately after 30 min in subjects who drank red wine and green tea. Similar to pili pomace drink, these beverages are rich in anthocyanins and low molecular weight flavanols, respectively (Serafini et al., 2000; Duthie et al., 1998).

FRAP levels were significantly higher in untreated compared to uricase-treated plasma (p<0.05). In the non-urate plasma, TAC was significantly (p<0.05) elevated within 30 min after consumption. The activity remained constant over the first hr, followed by a sharp rise in the 2^nd^ hr. TAC gradually depleted afterwards, although it remained above basal level until the 4^th^ hr. With regard to untreated plasma, changes in TAC determined within the 1^st^ hr were not statistically significant (p>0.05). TAC level peaked in the 2^nd^ hr and returned toward basal within 4 hr.

**Table 3:** Polyphenol content of pili pomace suspension

**Content**	** Supernatant (g/100ml)**		** Residue (g/100g)**	
	**Heated**	**Unheated**	**Heated**	**Unheated**
Total phenolics (GAE)	0.18+0.00	0.05+0.01	9.29+0.11	10.07+0.16
Total flavonoids (CE)	73.11+6.83	24.26+1.21	5.18+0.04 ^NS^	5.87+0.31^ NS^
Condensed tannins (CE)	30.70+7.83	not detected	8.01+0.16	11.10+0.11
Total anthocyanins (C3G)	0.03+0.00	0.02+0.00	0.08+0.01	0.14+0.01

Mean +SEM (n=3). Paired t-test comparisons between heated and unheated samples. NS - not significant (p>0.05).

**Table 4:** Antioxidant activity of pili pomace suspension

**Assay**	**Supernatant (µmol TE/L)**		**Residue (µmol TE/g)**	
	**Heated**	**Unheated**	**Heated**	**Unheated**
DPPH	13375.97+96.13	4253.19+125.36	985.55+59.76^NS^	1001.23+67.82^NS^
ABTS	21059.72+300.46	7865.28+431.22	1470.09+55.16^NS^	1543.39+39.1^NS^
FRAP	6688.24+66.79	1306.58+35.33	412.99+10.03	455.7+1.77

Mean +SEM (n=3). Paired t-test comparisons between heated and unheated samples. NS - not significant (p>0.05).

A parallel increase in polyphenol concentration and non-urate plasma TAC was observed 30 min post-consumption. At this time point, a marked increase (8.5%) in plasma polyphenols resulted in a 20% rise in TAC. Although data seemed to be suggestive of an association, it failed to demonstrate statistical significance (r = -0.296, p=0.407). It should be noted that both plasma polyphenol and non-urate plasma TAC remained above baseline values 4 hr after intake. It is uncertain how long this effect can be sustained since the experiment was terminated at the 4^th^ hr. Nonetheless, our data demonstrated that single intake of polyphenol-rich pili pomace infusion resulted to an improvement in antioxidant status.

## Discussion

Heating is a widely used process in food preparation and preservation -; But, it can lead to alterations in food composition, generally resulting in greater nutrient losses. Thus, the influence of thermal treatment on polyphenol content and antioxidant activity of pili pomace suspension was examined in the present study. Comparison between the heated and unheated supernatants showed that antioxidant activity significantly increased after heating. Likewise, similar effect was observed in polyphenol content. Heating increased total phenolics, flavonoids, condensed tannins and anthocyanins by 3.6, 3.0, 30.0 and 1.5 - fold, respectively. Antioxidant activity of heated supernatant increased by 3.1, 2.7, and 5.1 - fold as determined by DPPH, ABTS and FRAP methods, respectively. These results were consistent with the study of Choi et al. (2006) ▶ who reported that heat treatment enhanced antioxidant activity which corresponded to an increase in phenolic concentration. It is possible that heat breaks down plant cell walls thereby effectively releases entrapped phenolic compounds from the structural matrix. Data also indicates that phenolics extracted from pili pomace are heat stable. Additionally, it is also postulated that phenolics contained in the pili pomace are composed of the soluble or extractable type. These phenolics are characterized by low and intermediate molecular mass which are readily extracted bypolar solvents such as water (Saura-Calixto et al., 2007 ▶).

On the other hand, an opposite trend was observed for the residue. Losses in total phenolics, flavonoids, condensed tannins and anthocyanins were 7.8, 11.8, 27.8 and 42.9 %, respectively. Reductions in antioxidant activity were 1.6, 4.8 and 9.4 % as assessed by DPPH, ABTS and FRAP methods. The liberation of bound phenolics from the solid residue and its subsequent dissolution in the supernatant fraction could explain the reduction of phenolics and antioxidant activity noted in the heated residue.

Knowledge of bioavailability is vital in understanding the biological relevance of polyphenols. Bioavailability differs among polyphenols (Manach et al., 2004 ▶). Findings suggest that polyphenols derived from pili pomace are rapidly absorbed into the circulation. Maximum level was achieved between 30 and 60 min post-ingestion. Considering this abrupt postprandial effect, it can be speculated that the gastroduodenal junction is the main absorption site for these polyphenols. As stated earlier, soluble phenolics comprise the majority of the polyphenols present in pili pomace. These soluble phenolics, mostly phenolic acids and anthocyanins, are known to be quickly absorbed in the stomach and small intestine. 

Elevated plasma polyphenol levels were also reported after an acute intake of liquids such as red wine (Serafini et al., 1998 ▶), beer (Ghiselli et al., 2000a ▶), whisky (Duthie et al., 1998 ▶) and tea (Leenen et al., 2000 ▶). Similarly, Cassidy et al. (2006) ▶ demonstrated that compared to a solid matrix, soy isoflavones in a liquid matrix, like soy milk, show a more rapid rate of absorption and greater peak plasma concentrations. 

Furthermore, plasma polyphenol levels remained significantly higher relative to baseline throughout the entire duration of the trial (4 hr; p<0.05). This prolonged presence in the plasma can be explained by the ability of polyphenols and their metabolites in binding to plasma protein, primarily albumin. Affinity to albumin varies among polyphenols and is strongly dependent on their chemical structure. Consequently, the rate of elimination as well as cellular and tissue uptake of polyphenols is influenced by the degree of binding to albumin (Manach et al., 2004 ▶). The rate of elimination of pili polyphenols is seemingly low. Persistence of polyphenols in plasma may likely reflect their capability to sustain their biological activity *in vivo*. 

Oxidative stress arises when there is an imbalance between oxidants and antioxidants in the system, inducing a shift towards the oxidative potential (Serafini and Del Rio, 2004 ▶). Total antioxidant capacity (TAC) is a sensitive and reliable marker to study oxidative stress *in vivo*. In addition, plasma antioxidant capacity can be an indirect measure of phenolic bioavailability following ingestion of foods (Ghiselli et al., 2000b ▶). The ferric reducing antioxidant potential (FRAP) is an analytical method for TAC determination. This assay measures the ability of plasma antioxidants to reduce ferric to ferrous ions leading to the formation of colored ferrous-tripyridyltriazine complex (Serafini and Del Rio, 2004 ▶). Uric acid (UA) is a powerful antioxidant and accounts for ~60% of the TAC in plasma (Modun et al., 2008 ▶). Thus, interference due to UA must be corrected. This was done in this study by adding uricase to destroy UA followed by measurement of remaining TAC. Modun et al. (2008) ▶ also reported lower FRAP plasma values after uricase treatment. This observation suggests possible overestimation of TAC values if uric acid is not eliminated prior to FRAP analysis. Upon removal of uric acid, which is known to be a major contributor to TAC in FRAP, the actual contribution of dietary polyphenols to TAC became readily apparent.

The concerted effects of various phenolic compounds namely flavonoids, tannins and anthocyanins present in pili pomace drink, may have imparted TAC. Interestingly, maximum FRAP level was achieved at 2 hr despite the significant reduction in plasma total polyphenols found at this time point (p<0.05). Pearson correlation analysis showed that plasma polyphenol concentration was significantly and negatively correlated with TAC (r = -0.0775, p= 0.009) at this time-point. According to Manach et al. (2004) ▶, absorption can take place only after polyphenols have been hydrolysed either by enzymes in the intestines or by microorganisms in the colon. During the process of absorption, polyphenols undergo structural modifications via conjugation mechanisms – methylation, sulfation and glucuronidation, which occur in the small intestines and liver. As a result of the conjugation process, various metabolites are formed which consequently lowers the polyphenol level in the blood. In fact, several metabolites can be produced from a single polyphenol (D’Archivio et al., 2010 ▶). Ghiselli et al. (2000b) ▶ also pointed out that metabolization and biotransformation lead to differences in phenolic profile found in plasma and the original food. Furthermore, it has been observed that the enhancement of plasma antioxidant capacity is not entirely attributed to intact parent polyphenols since their amount is often low to elicit the effect. Phenolic metabolites also account for the increase in plasma antioxidant capacity (Scalbert and Williamson, 2000 ▶). Given this premise, it is possible that unidentified phenolic metabolites may have been responsible for the positive effect on systemic antioxidant status observed in this study. These metabolites, which are chemically different from their native counterpart, were not detected nor measured by spectrophotometric method used in this study. Moreover, it has been reported that some of these metabolites are more abundant in plasma and possess greater antioxidant potential than their parent compound (Scalbert and Williamson, 2000 ▶). Further work is warranted to elucidate phenolic metabolites from pili pomace using more advanced instrumentation to quantify their contribution to antioxidant activity *in vivo*. 

Pili pomace is a source of bioavailable polyphenols with corresponding antioxidant property, *in vivo*. An acute consumption of thermally processed pili pomace drink can modulate plasma antioxidant and polyphenols in humans. 

It is suspected that metabolites other than parental phenolic compounds might have also contributed to the antioxidant activity observed in this study. Future studies should be done on the unidentified metabolites of pili, in order to evaluate their contribution to plasma antioxidant potential. This study provides basis for the design of long-term dietary intervention trials that are necessary to fully understand the role of pili pomace in human nutrition.
